# Activation of the TGF-β1/Smads/α-SMA pathway is related to histological and functional changes in children with neurogenic bladder

**DOI:** 10.1038/s41598-022-13470-0

**Published:** 2022-06-03

**Authors:** Xinghuan Yang, Qingsong Pu, Yibo Wen, Yi Zhao, Junkui Wang, Pengchao Xu, Yuan Ma, Erpeng Liu, Lei Lv, Jian Guo Wen

**Affiliations:** 1 Department of Pediatric Urodynamic Center, Henan Joint International Pediatric Urodynamic Laboratory, The First Affiliated Hospital of Zhengzhou University, Zhengzhou, 450052 China; 2 Henan Joint International Pediatric Urodynamic Laboratory, Zhengzhou, China; 3 Urinary Bladder Structure and Function Reconstruction Laboratory (Henan Developing and Reform Committee), Zhengzhou, China

**Keywords:** Diseases, Medical research, Urology

## Abstract

This research is to investigate the expression of the TGF-β1/Smads/α-SMA pathway and its effect on bladder histology and function in children with neurogenic bladder (NB). The bladder specimens from 10 children with NB and 8 children with vesicoureteral junction obstruction were collected into the NB and control groups. The expression of TGF-β1, Smad2, Smad3, Smad4, Smad6, α-SMA, fibronectin, collagen I and collagen III in bladder tissues was detected. In addition, the histological characteristics of the bladder were evaluated. A preoperative urodynamic study was performed on all children with NB. We analysed the correlations among the expression of the marker protein a-SMA in myofibroblasts, effector cells of the pathway, and bladder function parameters. Compared with those in the control group, the expression of TGF-β1, Smad2, Smad3, Smad4, α-SMA, fibronectin, collagen I and collagen III was significantly increased in the NB group, while the expression of Smad6 was decreased (*p* < 0.01). HE and Masson staining in the NB group showed increased collagen levels and hypertrophy of smooth muscle cells. Children with NB had a low bladder volume ratio (BVR), low compliance (△C) and high maximum bladder pressure, low maximum flow rate, large postvoid residual volume, low bladder contraction index and low bladder voiding efficiency. The expression of α-SMA was negatively correlated with the BVR (r =  − 0.7066, *P* = 0.0223) and △C (r =  − 0.6516, *P* = 0.0412). We conclude that the TGF-β1/Smads/α-SMA pathway is activated in the bladder tissue of children with NB and may be involved in the processes causing histological and functional changes.

## Introduction

Neurogenic bladder (NB) is well known to be caused by neurological abnormalities controlling the lower urinary tract, of which the most common cause in children is spinal dysplasia. This leads to bladder and urethral dysfunction, including overactive bladder, detrusor sphincter dyssynergia, and underactive bladder or acontractile detrusor^1^. These abnormalities will cause the bladder to expel urine from the body through compensation mechanisms, such as increased capacity or increased contractility^2^. As the disease progresses, the bladder remodels and bladder function deteriorates further, resulting in decreased bladder capacity, poor compliance and increased residual urine^3,4^. These changes damage the anti-reflux mechanism of the vesicoureteral junction and result in urine reflux, which can cause upper urinary tract dilation, kidney tissue damage and even kidney failure, thereby increasing the risk of death^4,5^. The process of bladder decompensation is mainly thought to be related to bladder remodelling, especially bladder fibrosis worsening^6,7^. Therefore, preventing histological changes in the bladder may effectively delay disease progression, but the specific molecular mechanism underlying this process is still unclear.

TGF-β1 has been shown to be an important factor involved in histological changes in various organs^8^. During fibrosis progression in these tissues, TGF-β1 binds to its receptor and participates in intracellular signal transduction by interacting with the downstream Smad proteins, regulating the expression of corresponding genes, and increasing myofibroblast numbers. Myofibroblasts produce excessive extracellular matrix (ECM), including collagen and fibronectin (FN), thus causing fibrosis^9,10^. Results obtained with the rat model of spinal cord transection we built and those of other rat experiments related to bladder outlet obstruction found that TGF-β1/Smad pathway members are highly expressed in the bladder tissue and may mediate changes in the structure and function^11,12^. Whether this pathway is also related to changes in the bladders of children with NB is unknown. In the present study, we compared the pathological characteristics and expression of pathway-related proteins in bladder specimens from children with and without NB and analysed the correlations between the expression of myofibroblast markers (α-SMA) and bladder function parameters to explore the effect of this pathway on bladder histology and function. The results of this study may provide an important reference for understanding the mechanism underlying changes in NB.

## Results

### Expression of pathway

**Figure 1:**
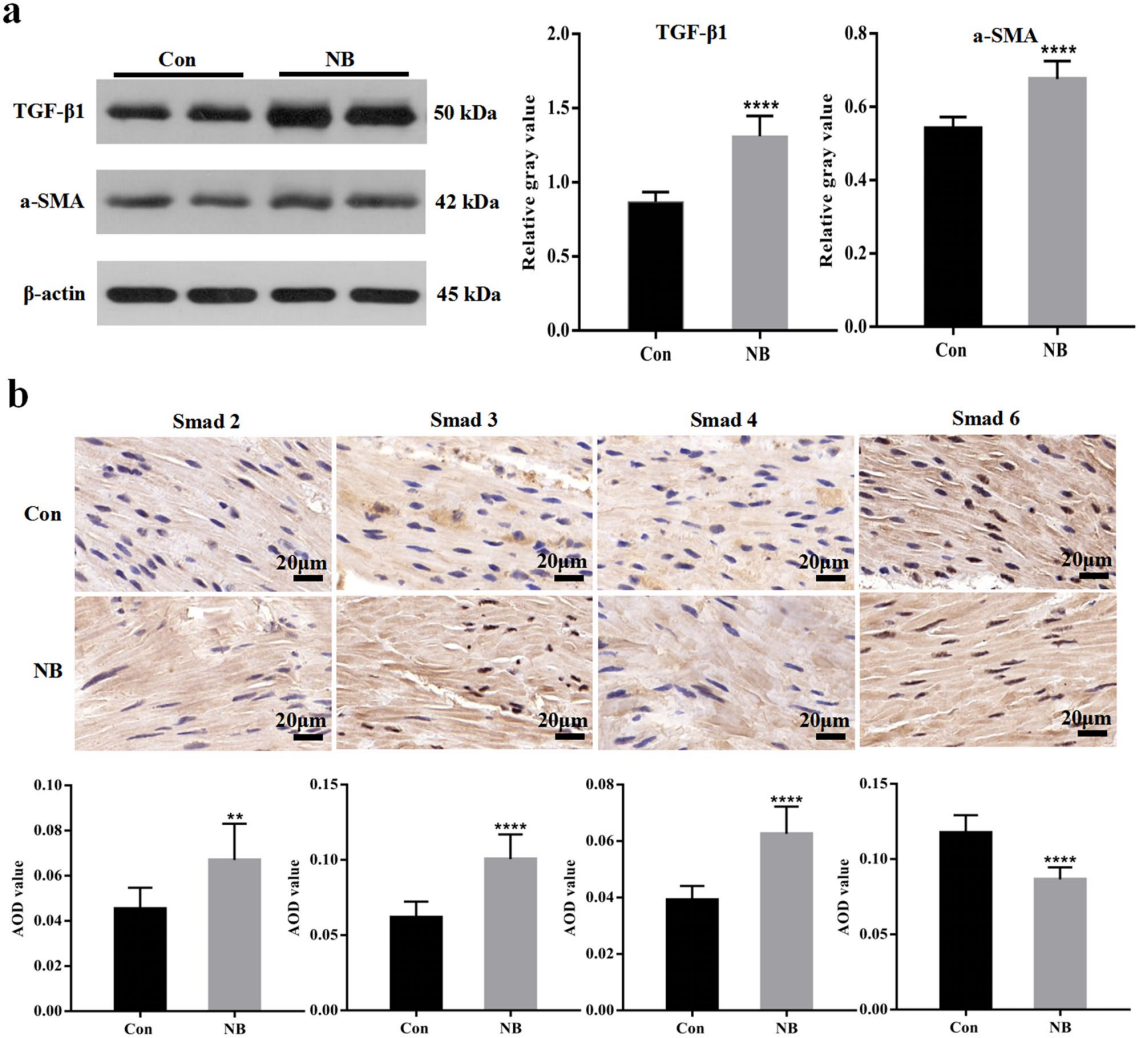
Expression of TGF-β1, Smad2, Smad3, Smad4, Smad6 and α-SMA in the NB and control groups: (**a**) Western blot analysis of TGF-β1 and α-SMA. The gray value ratios of TGF-β1 and α-SMA to β-actin between the two groups were compared. Representative western blots were cropped from the same blots. Original blots are presented in Supplementary material. (**b**) Immunohistochemical staining of Smad2, Smad3, Smad4, and Smad6. The brownish-yellow area in the figure indicates that positive staining for the target protein. The AOD values of each Smad protein were compared between the two groups. The bars indicate 20 μm. The data are shown as the means ± SD. **p < 0.01; ***p < 0.001; ****p < 0.0001 versus the control. Student’s t test. AOD, average optical density.

Significant differences were observed in the pathway expression in the bladder tissues of the NB and control groups. Western blot analysis of TGF-β1 and α-SMA showed that the expression of TGF-β1 and α-SMA in the bladder tissue of the NB group was significantly higher than that in the bladder tissues of the control group (Fig. 1a). Immunohistochemical staining showed that compared with the control group, the expression of Smad2, Smad3, and Smad4 was increased in the NB group, while that of Smad6 was decreased (Fig. 1b).

### Histological change

**Figure 2:**
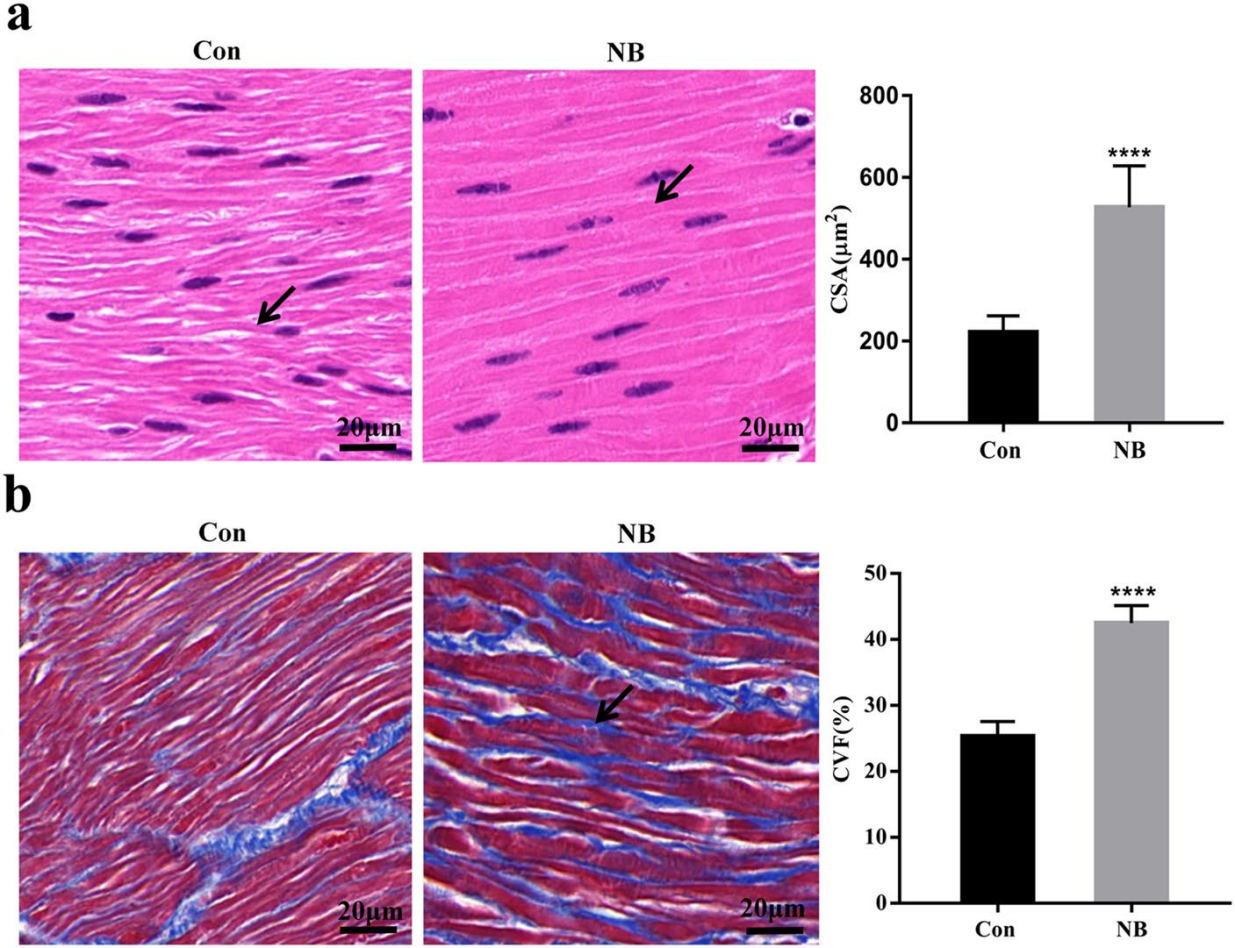
HE and Masson staining of bladder tissue in the NB group and control group: (**a**) HE staining of bladder tissue. The red area in the figure represents the detrusor cytoplasm, and the purplish-blue area represents the nucleus. The black arrow indicates detrusor cells. The CSA values of the two groups were compared. The bars indicate 20 μm. (**b**) Masson staining of bladder tissue. The blue area in the figure represents collagen fibres, and the red area represents muscle fibres. The white arrow indicates collagen fibres. The CVF values of the two groups were compared. The bars indicate 20 μm. The data are shown as the means ± SD. ****p < 0.0001 versus the control. Student's *t* test. CSA, mean cross-sectional area. CVF, Collagen volume fraction.

HE and Masson staining showed that the bladder tissues of the NB group had obvious histological changes. HE staining showed degeneration of detrusor muscle cells in the NB group, with abnormal morphology, cell hypertrophy, increased cytoplasmic ratios and decreased nucleocytoplasmic ratios (Fig. 2a). Masson staining showed a large number of disordered and diversified collagen fibres between detrusor muscle cells in the NB group, which caused a significant increase in cell spacing. Moreover, the collagen volume fraction (CVF) value of the NB group was significantly higher than that of the control group (Fig. 2b).

### Expression of fibrosis related proteins

**Figure 3:**
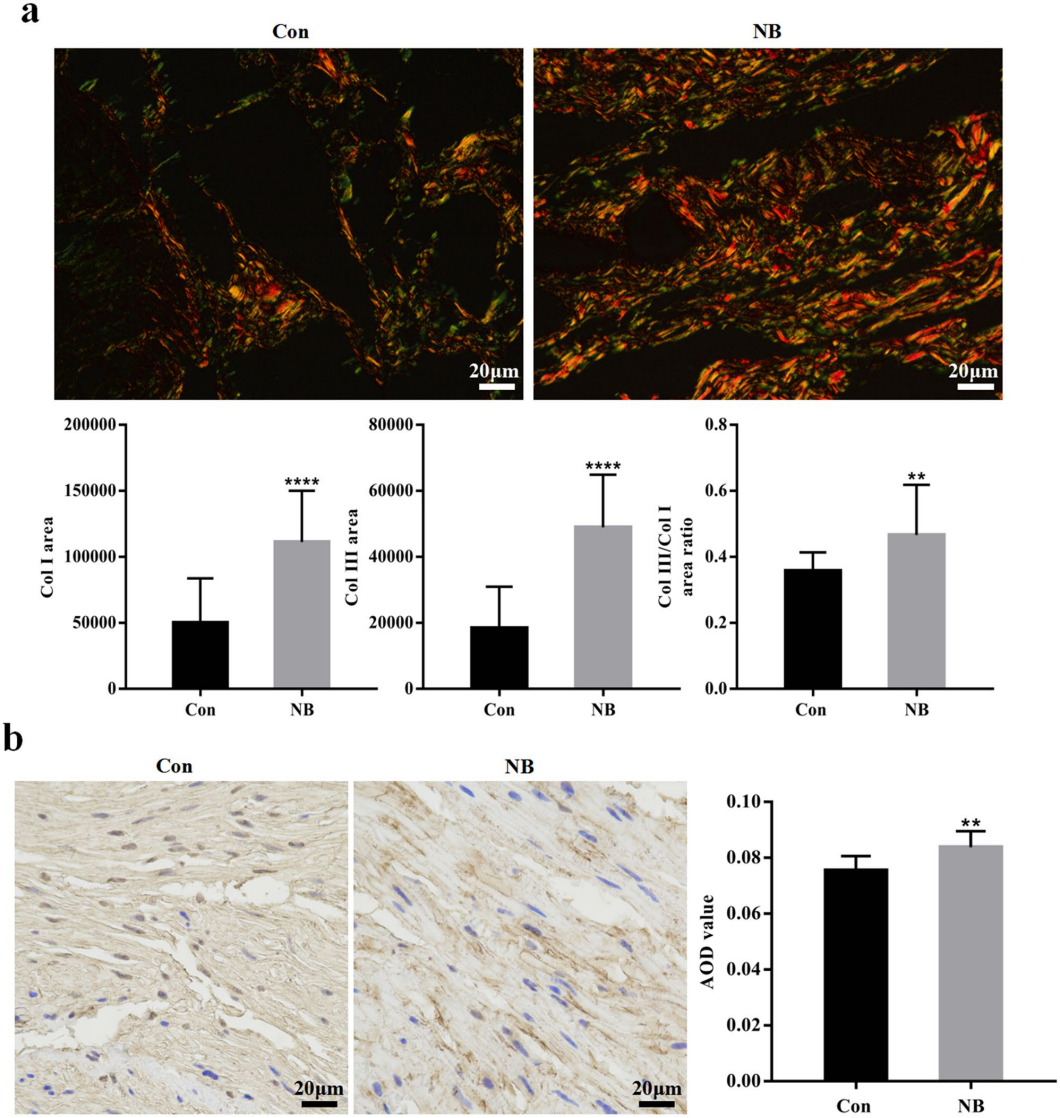
Specific staining of fibrosis-related proteins: (**a**) Sirius red staining of the two groups observed with a polarized light microscope. The orange or bright red crude fibres in the picture indicate Col I and the fine green fibres indicate Col III. The areas of collagen I and collagen III and the area ratios of collagen III to collagen I were compared between the two groups. (**b**) Immunohistochemical staining of FN. The brownish-yellow area in the figure indicates positive FN staining. The AOD values were compared between the two groups. The bars indicate 20 μm. The data are shown as the means ± SD. **p < 0.01; ****p < 0.0001 versus the control. Student’s *t* test. AOD, average optical density.

Specific staining of fibrosis-related proteins revealed higher expression in the NB group than in the control group. Sirius Red staining showed that the expression of Col I and Col III in the bladder tissue of the NB group was increased, and the area ratio of Col III to Col I was increased compared with that in the control group (Fig. 3a). Immunohistochemical staining showed that the average optical density (AOD) value of FN was higher in the NB group than in the control group (Fig. 3b).

### Correlation between the expression of α-SMA and bladder function parameters

**Figure 4:**
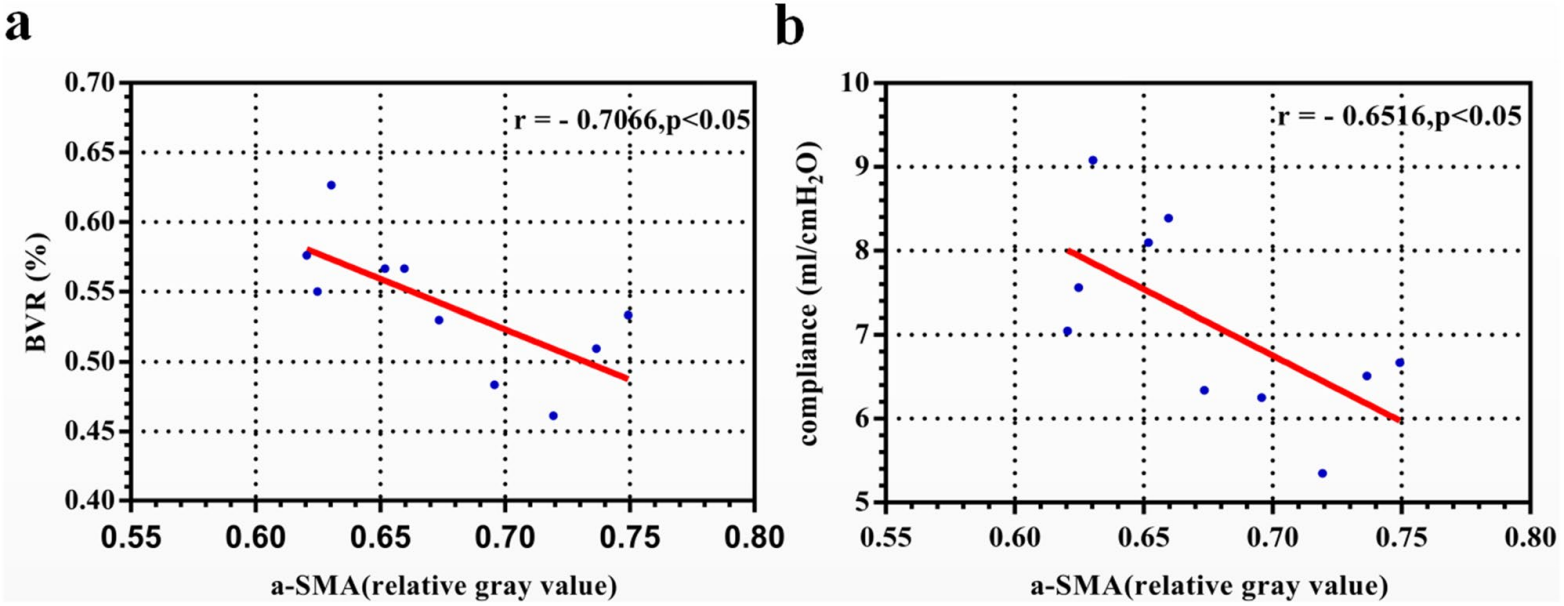
Correlation between the expression of α-SMA and BVR (**a**) or △C (**b**).

After summarizing the bladder function parameters of 10 children with NB, we found that the maximum cystometric capacity (MCC), bladder volume ratio (BVR), compliance (△C), maximum flow rate (Qmax), bladder contraction index (BCI), and bladder voiding efficiency (BVE) values were lower than the normal range, while the maximum detrusor pressure during filling (maxPdet) and postvoid residual (PVR) values were significantly higher (Table 1)^13–17^. Analysis of the correlation between the expression of α-SMA and bladder function parameters revealed that the expression of α-SMA was negatively correlated with the BVR (r =  − 0.7066, *p* < 0.05) and △C (r =  − 0.6516, *p* < 0.05) (Table 1, Fig. 4).

**Table 1:** Correlation between the expression of a-SMA and bladder function parameters.

Parameter	Mean ± SD	r	*p* value
MCC (ml)	127.00 ± 39.08	− 0.3172	0.3719
BVR (%)	54.04 ± 4.82	− 0.7066	0.0223*
△C (ml/cmH_2_O)	7.13 ± 1.14	− 0.6516	0.0412*
MaxPdet (cmH_2_O)	83.50 ± 17.77	0.4216	0.2250
Qmax (ml/s)	2.74 ± 0.91	− 0.2244	0.5331
PdetQmax (cmH_2_O)	42.40 ± 6.70	− 0.3715	0.2905
BCI	57 ± 4.67	− 0.5791	0.0794
PVR (ml)	89.50 ± 33.71	− 0.3442	0.3301
BVE (%)	29.66 ± 11.48	0.2637	0.4617

The results are expressed as the mean ± SD. Correlations were assessed by the Spearman test.

MCC, maximum cystometric capacity; BVR, bladder volume ratio; △C, compliance; maxPdet, maximum detrusor pressure during filling; Qmax, maximum flow rate; PdetQmax, pressure at the peak flow rate; BCI, bladder contraction index; PVR, postvoid residual urine volume; BVE, bladder voiding efficiency.

*p < 0.05.

## Discussion

Various treatment methods, including medication and surgery, have been widely used in the treatment of NB, but their therapeutic effects remain unsatisfactory^18^. This may be because most treatments only deal with symptoms and cannot effectively prevent changes in bladder histology or function. The mechanism underlying this process is still unclear, making it difficult to find more effective treatment methods. Our research shows that the TGF-β1/Smads/α-SMA pathway is activated in patients with NB, and that this may be related to changes in bladder histology and function.

TGF-β1 is an important cytokine that is related to many physiological processes, including differentiation, proliferation, immune regulation, and tissue repair^8^. It binds to heterotetrameric receptors on the cell surface to activate and phosphorylate Smads, a key downstream regulators in the pathway. Thereafter, signals are transmitted to the nucleus to activate the expression of corresponding target genes^19^. In the process of tissue fibrosis, phosphorylated Smad2 and Smad3 and the common regulator Smad4 form a heterogeneous complex to promote fibrosis development, while Smad6 plays a major negative regulatory role in this process^20^. Myofibroblasts are the main effector cells of this pathway. Studies suggest that an increase in the number of myofibroblasts will cause excessive ECM accumulation and tissue fibrosis^21,22^. The expression of the α-SMA protein, a myofibroblast marker, increases upon the induction of tissue fibrosis^23^. Few studies have investigated the mechanism by which this pathway regulates changes in the bladders of children with NB. Some studies reported that this pathway may play a role in the process of bladder fibrosis in NB and partial bladder outlet obstruction (PBOO) rat models, and the use of sodium tanshinone IIA sulfonate (STS) was shown to effectively inhibit the occurrence of bladder fibrosis mediated by this pathway in PBOO rats^11,24,25^. Our study first found that the expression of TGF-β1, Smad2, Smad3, Smad4, and α-SMA was increased, while that of Smad6 was significantly decreased in the bladders of children with NB (Fig. 1). This result indicates that this pathway is activated and that myofibroblasts are increased in patients with NB. Because this process may mediate the occurrence of fibrosis, it is reasonable to speculate that the mechanism of fibrosis in NB is similar to that in other tissues and is mediated by this pathway. This discovery will provide an important basis for future research in this area.

Howard PS cultured human bladder smooth muscle cells (SMCs) with TGF-β1 and its antagonist in vitro and found that TGF-β1 not only increased the expression of type I and type III collagens but also caused detrusor muscle cell hypertrophy. Therefore, he concluded that TGF-β1 mediated not only fibrosis but also detrusor hypertrophy^26^. In this study, we found similar pathological changes through HE and Masson staining. Subjects with NB exhibited not only fibrosis but also detrusor hypertrophy (Fig. 2). These findings indicate that bladder remodelling in children with NB includes fibrosis and detrusor hypertrophy and that activation of the TGF-β1-mediated pathway may play an important role in this process. However, the subsequent intracellular signal transmission process and characteristics of TGF-β1-mediated detrusor hypertrophy are not clear and will be a direction of our future research.

The occurrence of tissue fibrosis is related to the deposition of collagen and fibronectin, both of which are the key indicators used to evaluate the degree of fibrosis. Studies have found that Col I and Col III are the main collagens involved in bladder fibrosis and are characterized by an increase in the ratio of type III to type I collagens^27^. Relative quantitative analysis of the Sirius red staining results revealed that the expression of Col III and Col I in the bladder tissue was increased as well as the ratio (Fig. 3a). This result was further explored and confirmed the change characteristics of collagen in NB. This result may serve as a good reminder to seek new diagnoses and interventions. FN was found to play a connecting role in the organization. Collagen is deposited between cells by binding to FN^28^. Herein, the expression of FN in the bladder tissue of the NB group was increased (Fig. 3b). These changes indicate that the process of fibrosis in NB is related to the increased expression of these two components. Controlling the production of these proteins by treatment may effectively prevent the development of bladder fibrosis.

Children with NB may have abnormal bladder functions, including detrusor overactivity, detrusor sphincter dyscoordination, and underactive bladder or acontractile detrusor, in the early stage of disease, and bladder function deteriorates as the disease progresses^3^. We subjected 10 children with NB to a preoperative urodynamic study (UDS) and found that the bladder function parameters were obviously abnormal. The results revealed low bladder compliance, low bladder capacity and high intravesical pressure at the filling phase. Weak bladder contractile function, a low urine flow rate and a large residual urine volume were observed during the voiding phase. These changes will prohibit the effective elimination of urine from the body and increase the risk of upper urinary tract damage^13^. This suggests that changes in bladder function should be prevented in a sufficient amount of time to reduce upper urinary tract damage.

However, the specific mechanism underlying bladder function changes is still unclear. It is currently thought to be related to abnormal innervation, increased ECM accumulation, and abnormal SMC function^2^. Our study found that the relative protein level of α-SMA protein was negatively correlated with the BVR and △C, indicating that the increase in myofibroblast numbers may have affected bladder function, possibly due to overexpression of extracellular stroma (ECM). Studies have shown that a large amount of ECM deposition increases the hardness of the tissue, thereby decreasing compliance. These changes decrease the bladder distensibility, thus reducing bladder capacity. At the same time, the increase in ECM accumulation results in the percentage of detrusor muscle in the tissue being decreased and the space between cells being increased. These changes may affect signal transmission and the number of cell-to-cell contacts, thereby resulting in reduced contractile function^29^. However, this study found that the expression of α-SMA was only correlated with changes in the BVR and △C and not with changes in bladder contraction. This result may have been due to the small sample size. In the future, we will expand the sample size to further explore its influence on bladder contraction.

Our research has some limitations. First, due to ethical constraints, we could not perform invasive UDS on children in the control group, and we thus obtained the ranges of normal bladder function parameters from related studies. Second, we did not explore the upstream signalling pathways that induce the activation of this pathway. It is currently believed that high pressure, hypoxia, and neurological abnormalities lead to subsequent changes in NB^2^. Therefore, the specific process from the occurrence of these inductions to the activation of the pathway is unclear. In the future, we will explore this process and intervene appropriately with antagonistic drugs. This will directly prevent the activation of the pathway, which may better prevent changes in NB. In addition, recent studies demonstrated that the anti-fibrosis factor miR-133a can effectively modulate detrusor cell hypertrophy and fibrosis induced by TGF-β1^30^. We plan to next use this approach in a rat model of spinal cord transection to explore its efficacy and provide a reference for future clinical applications.

In summary, this study found that TGF-β1/Smads/α-SMA pathway members are highly expressed in the bladders of children with NB. The histological and functional changes in NB may be related to the activation of this pathway. This result suggests that in the treatment of PNB, in addition to expanding the bladder capacity, reducing the pressure in the bladder, and protecting the upper urinary tract, new medicines should be developed to prevent the activation of this pathway in future studies.

## Methods

The Institutional Review Board of First Affiliated Hospital of Zhengzhou University approved this study. The legal guardian of all children were informed of the purpose of the study. They signed *written informed consent* and allowed us to conduct research on bladder specimens. All experiments were performed in accordance with relevant named guidelines and regulations.

Between December 2018 and April 2021, 10 children with NB (6 boys, 4 girls; age, 6.90 ± 2.60 years) undergoing ileocystoplasty were enrolled in the study. The inclusion criteria included 1) myelodysplasia and 2) video urodynamic study showing that the bladder volume was significantly reduced and that vesicoureteral reflux occurred. The control group included 8 children (4 boys, 4 girls; age, 6.38 ± 3.85 years) with vesicoureteral junction obstruction who underwent ureteral reimplantation due to vesicoureteral reflux. None of them had lower urinary tract symptoms or suffered from NB.

A full-thickness incisional bladder specimen (approximately 1.5 cm × 0.3 cm) was obtained from the edge of the surgical cut during the process of ileocystoplasty or ureteral reimplantation. The bladder specimens werre quickly washed three times with phosphate buffered saline solution (PBS) and then divided into two parts. One was stored at − 80 °C for western blot analysis, and the other was fixed with 10% neutral formalin for 24 h and then embedded in paraffin. The wax block was sliced into 5-µm-thick sections on a paraffin microtome (RM2016, Leica, Shanghai, China). The sections were then transferred onto glass slides and placed in a 40 °C incubator for 24 h.

### Western blotting

Proteins were extracted after grinding the specimens, and their concentrations were determined using the Pierce BCA Protein Assay Kit (Trans, Beijing, China). 20 µg of protein from each sample was loaded onto gels and separated by sodium dodecyl sulfate–polyacrylamide gel electrophoresis. The proteins were then transferred onto polyvinylidene fluoride (PVDF) membranes. To save material and facilitate subsequent experiments, the membranes were cut prior to hybridisation with antibodies. As the predicted molecular size of TGF-β1, α-SMA and β-actin were all between 40 and 55 kDa, they were detected by reprobing the same cut section of the membrane. The membranes were first incubated overnight with anti-α-SMA rabbit pAb (GB111364, Servicebio, Wuhan) in TBST with 5% nonfat milk. Next, they were incubated with the secondary antibody at room temperature for 1 h. The X-ray film was developed and fixed by the enhanced chemiluminescence method. Restore western blot stripping buffer (21,059, Thermo Scientific, Shanghai) was used to strip antibodies from membranes. The membranes were then incubated overnight with anti-TGF-β1 rabbit pAb (GB111876, Servicebio, Wuhan) in TBST with 5% nonfat milk. Followed by secondary antibody hybridization, chemiluminescence and antibody stripping as described above. Finally, the membranes were incubated overnight with anti-β-actin rabbit pAb (GB11001, Servicebio, Wuhan) in TBST with 5% nonfat milk. Followed by secondary antibody hybridization and chemiluminescence as described above. After scanning the X-ray film, the gray values of the bands were measured using a computer-assisted morphometric analysis system (Image-Pro Plus 6.0 software). β-actin was used as the internal reference. The protein expression was calculated based on the ratio of the gray value of the protein to β-actin.

### Immunohistochemical staining

The sections were dewaxed, washed, and placed in citrate antigen retrieval solution. H_2_O_2_-methanol (3%) was used to block endogenous peroxidase, and BSA-PBS (3%) was added to block the serum. The primary antibodies anti-smad2 rabbit pAb (GB11511, Servicebio, Wuhan), anti-smad3 rabbit pAb (PA5-85259, Thermo Fisher Scientific, Shanghai), anti-smad4 rabbit pAb (PA5-34806, Thermo Fisher Scientific, Shanghai), anti-smad6 rabbit pAb (PA5-99401, Thermo Fisher Scientific, Shanghai), and anti-FN rabbit pAb (PA5-29578, Thermo Fisher Scientific, Shanghai) were added to the sections, which were then incubated at 4 °C overnight. The tissue was then covered with a secondary antibody (GB23303, Servicebio, Wuhan) and incubated at room temperature for 1 h. The sections were developed with DAB colour developing solution, and the nuclei were stained with haematoxylin solution. The images were observed and photographed using a high-power light microscope (Nikon Eclipse E100, Nikon, Japan). Positive protein expression is shown as a brownish-yellow color in the figure. In each section, five random fields were imaged at high magnification (400×). The integral optical density (IOD) and areas of these regions were measured by Image-Pro Plus 6.0. The average optical density (AOD) value was calculated as follows: AOD = IOD/area.

### HE and Masson staining

The sections were dewaxed and washed before HE and Masson staining. The images were observed and photographed by a high-power light microscope. In HE staining, the cytoplasm is stained red, and the nucleus is stained purplish-blue. In each section, five random fields were imaged at high magnification (400×). The transnuclear cross-sectional area of SMCs was quantified with Image-Pro Plus 6.0. The mean cross-sectional area (CSA) for all fields was calculated. Masson staining showed that the collagen fibers were blue and that the muscle fibers were red. In each section, five random fields were imaged at high magnification (400×). The average collagen area and area of the total field were quantitatively analyzed by Image-Pro Plus 6.0. The collagen volume fraction (CVF) was calculated as follows: CVF = average collagen area/area of total field × 100%.

### Sirius red staining

Sections were dewaxed and washed, after which the cells were placed in Sirius Red solution for 8 min. The cells were observed under a polarized light microscope, and collagen I (Col I) was stained orange or bright red, while collagen III (Col III) was stained green. In each section, five random fields were imaged at high magnification (400×). Image-Pro Plus 6.0 software was used to measure the areas of Col III and Col I. The area of the Col III to Col I ratio was calculated.

### Urodynamic study

All children with NB were subjected to a urodynamic study (UDS) in an awake state before surgery. The bladder function parameters assessed included the maximum cystometric capacity (MCC), bladder volume ratio (BVR), compliance (△C), maximum detrusor pressure during filling (maxPdet), maximum flow rate (Qmax), detrusor pressure at maximum flow rate (PdetQmax), postvoid residual (PVR), bladder contraction index (BCI), and bladder voiding efficiency (BVE), where BVR = MCC/EBV^13^. BCI = PdetQmax + 5Qmax^14^. and BVE = maximum voided volume/MCC^15^. The UDS was completed by the same physician for all children. In addition, the correlations between the expression of α-SMA and the above parameters were analyzed.

### Statistical analysis

SPSS software version 17.0 software (IBM) was used to analyze the data. The results are expressed as the means ± SD. Data were compared with the t-test between two groups. Correlations were assessed by the Spearman test. *P* < 0.05 was considered to be significantly different.

### Ethics approval

The study was approved by the Institutional Review Board of First Affiliated Hospital of Zhengzhou University (Henan,China) and was in accordance with *the principles of* the 1964 Declaration of Helsinki and its later amendments or comparable ethical standards.

### Consent to participate

The legal guardian of all children were informed of the purpose of the study. Written informed consent was obtained from them.

## Supplementary Information

Supplementary Information 1.

Supplementary Information 2.

## Data Availability

The datasets generated during and/or analysed during the current study are available in the figshare repository, https://figshare.com/s/a2ddc6049b738f4cb030.

## Code availability

All software applications used during this study are included in the manuscript.
