# Supplementary material for: Activation of the TGF-β1/Smads/α-SMA pathway is related to histological and functional changes in children with neurogenic bladder
Source: Sci Rep. 2022 Jun 3;12:9285. doi: 10.1038/s41598-022-13470-0 (PMC9166803; doi:10.1038/s41598-022-13470-0)
Supplement: Supplementary file 1 — Supplementary Information 1. [file 41598_2022_13470_MOESM1_ESM.pdf]

Activation of the TGF-β1/Smads/α-SMA pathway is related to histological and functional changes in children with neurogenic bladder

Xinghuan Yang[1,2,3], Qingsong Pu[1,2,3], Yibo Wen[1,2,3], Yi Zhao[1,2,3], Junkui Wang[1,2,3], Pengchao Xu[1,2,3], Yuan Ma[1,2,3], Erpeng Liu[1,2,3], Lei Lv[1,2,3] & Jianguo Wen[1,2,3,*]

[1]Pediatric Urodynamic Centre. First Affiliated Hospital of Zhengzhou University, Zhengzhou, China

[2]Henan Joint International Pediatric Urodynamic Laboratory, Zhengzhou, China

[3]Urinary bladder structure and function reconstruction laboratory (Henan Developing and Reform Committee), Zhengzhou, China

All the Product Information in the supplementary file is to prove that all the antibodies used in this study are specific to the target protein.All product information is the original provided by Company.

Anti -TGF-β1 Rabbit Polyclonal Antibody

GB111876 100μL -20℃

Product Information

**Description**	TGF beta 1 rabbit polyclonal antibody
**Protein full name**	Transforming growth factor beta-1 proprotein
**Synonyms**	CED, DPD1, LAP, TGF beta 1, tgf beta1, TGFB, TGFB1, TGFbeta, tgfbeta1, tgf-beta1, TGFβ, TGFβ1, TGF-β1
**Immunogen**	KLH conjugated Synthetic peptide corresponding to Mouse TGF beta 1
**Isotype**	IgG
**Purity**	Affinity purification
**Predicted MW.**	44 kDa
**Observed MW.**	50 kDa
**Uniprot ID**	P01137

Applications

Applications	Species	Dilution	Positive Sample
WB	Human	1: 500-1: 1000	HeLa, A549, K562, HEK293

Background

TGFB, also named as LAP and TGFB1, is a multifunctional peptide that controls proliferation, differentiation, and other functions in many cell types. TGFB acts synergistically with TGFA in inducing transformation. It also acts as a negative autocrine growth factor. Dysregulation of TGFB activation and signaling may result in apoptosis. Many cells synthesize TGFB and almost all of them have specific receptors for it. TGFB positively and negatively regulates many other growth factors. It plays an important role in bone remodeling as it is a potent stimulator of osteoblastic bone formation, causing chemotaxis, proliferation and differentiation in committed osteoblasts. It is highly expressed in bone.

Storage

**Storage**	Store at -20 ℃ for one year. Avoid repeated freeze/thaw cycles.
**Storage Buffer**	PBS with 0.02%sodium azide,100 μg/ml BSA and 50% glycerol.

NOTE:1.This product is intended for research only.
2.This product is recommended to dilute with the Primary Antibody Dilution Buffer.

Images

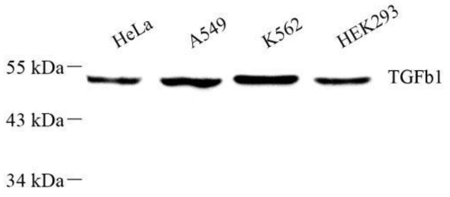

Western blot analysis of TGFb1 (GB111876) at dilution of 1: 500

Anti - a-S M A Rabbit Polyclonal Antibody

GB111364 100µL -20℃

Product Information

**Description**	alpha smooth muscle Actin rabbit polyclonal antibody
**Protein full name**	Actin, aortic smooth muscle
**Synonyms**	Actin, aortic smooth muscle, Alpha-actin-2, Cell growth-inhibiting gene 46 protein, ACTA2, ACTSA, ACTVS, GIG46, SMA, a-sma, sma
**Immunogen**	KLH conjugated Synthetic peptide corresponding to Mouse α smooth muscle actin
**Isotype**	IgG
**Purity**	Affinity purification
**Subcellular location**	Cytoplasm
**Predicted MW.**	42 kDa
**Observed MW.**	42 kDa
**Uniprot ID**	P62736, P62737, P62738

Applications

Applications	Species	Dilution	Positive Sample
WB	Human, Mouse, Rat	1: 1000-1: 2000	heart, liver
IHC/IF	Human, Mouse, Rat	1: 300-1: 1000	breast cancer, heart, liver, lung, lung cancer, brain, stomach
IEM	Mouse	1: 50	aorta

Background

Alpha-actin-2 also known as actin, aortic smooth muscle or alpha smooth muscle actin (α -SMA, SMactin, alpha-SM-actin, ASMA). Actin alpha 2, the human aortic smooth muscle actin gene, is one of six different actin isoforms which have been identified. Actins are highly conserved proteins that are involved in cell motility, structure and integrity. Alpha actins are a major constituent of the contractile apparatus.Alpha-smooth muscle actin (α -SMA) is commonly used as a marker of myofibroblast formation.

Storage

**Storage**	Store at -20 ℃ for one year. Avoid repeated freeze/thaw cycles.
**Storage Buffer**	PBS with 0.02%sodium azide,100 μg/ml BSA and 50% glycerol.

NOTE:1.This product is intended for research only.
2.This product is recommended to dilute with the Primary Antibody Dilution Buffer.

Images

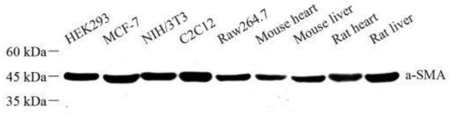

Western blot analysis of alpha smooth muscle Actin (GB111364) at dilution of 1: 1000

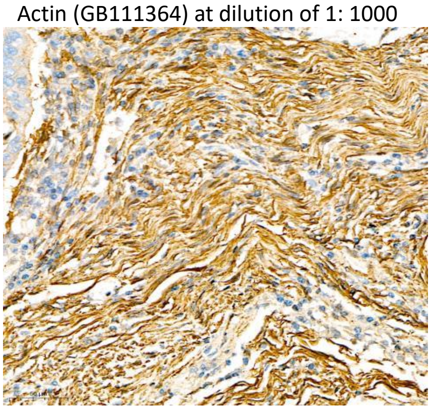

Immunohistochemistry analysis of paraffin-embedded human lung cancer using Actin (GB111364) at dilution of 1: 1000

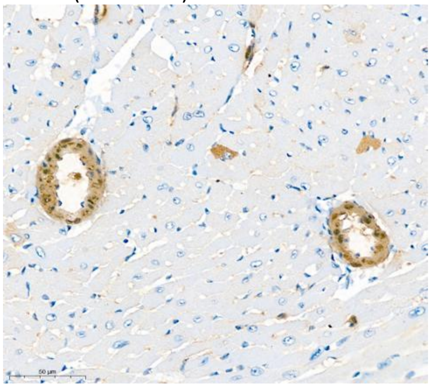

Immunohistochemistry analysis of paraffin-embedded mouse heart using Actin (GB111364) at dilution of 1: 1000

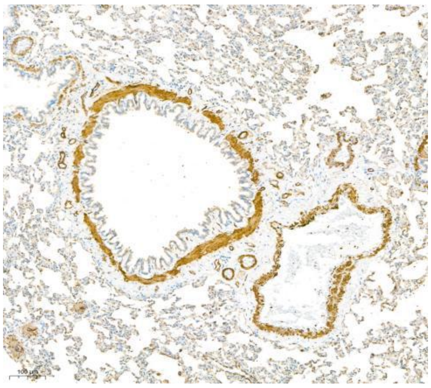

Immunohistochemistry analysis of
paraffin-embedded mouse lung using
Actin (GB111364) at dilution of 1: 1000

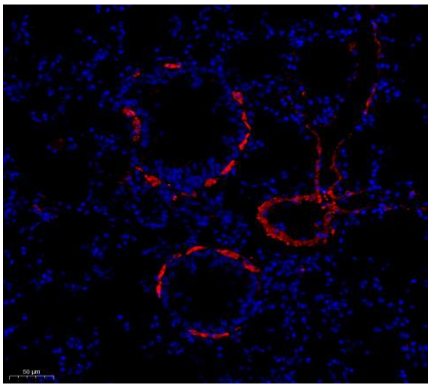

Immunofluorescent analysis of
paraformaldehyde-fixed mouse lung using
Actin (GB111364) at dilution of 1: 300

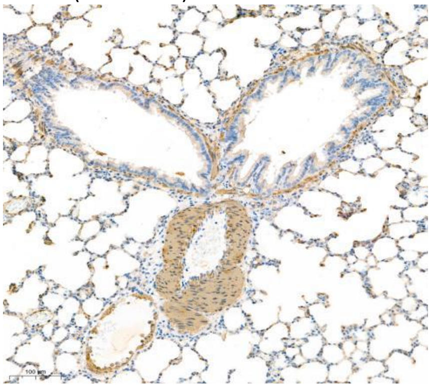

Immunohistochemistry analysis of
paraffin-embedded rat lung using
Actin (GB111364) at dilution of 1: 1000

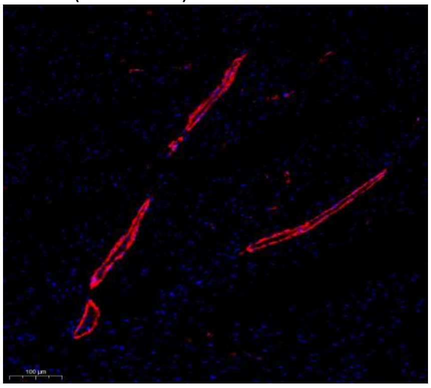

Immunofluorescent analysis of
paraformaldehyde-fixed rat heart using
Actin (GB111364) at dilution of 1: 300

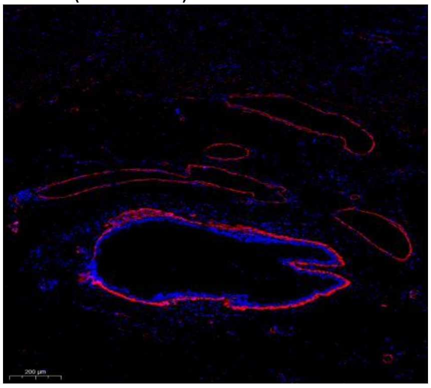

Immunofluorescent analysis of
paraformaldehyde-fixed human breast cancer
using Actin (GB111364) at dilution of 1: 300

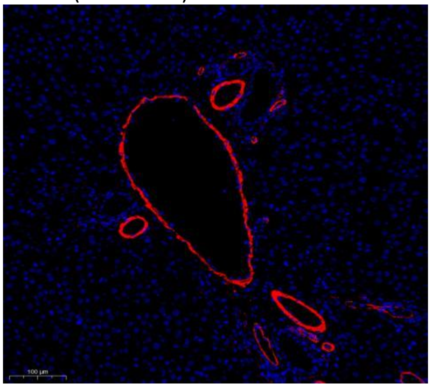

Immunofluorescent analysis of
paraformaldehyde-fixed rat liver using
Actin (GB111364) at dilution of 1: 300

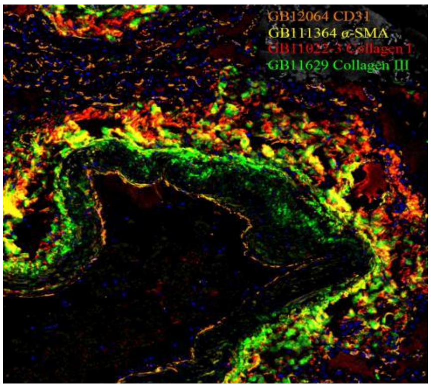

Immunofluorescent analysis of paraformaldehyde-fixed human para-cancer lung using COLIII (GB11629) (green) + Collagen I (GB11022-3) (red) + Actin (GB111364) (yellow) + CD31 (GB12064) (orange) at dilution of 1: 300

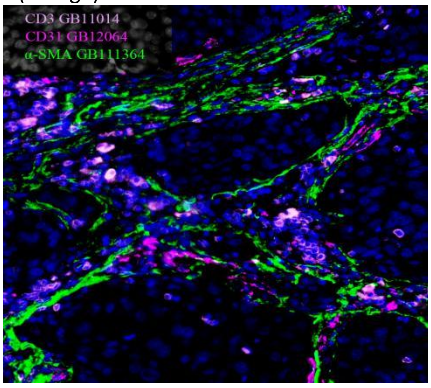

Immunofluorescent analysis of paraformaldehyde-fixed human lung cancer using Actin(GB111364) (green) + CD31(GB12064) (spred) + CD3(GB11014) (pink) at dilution of 1: 300

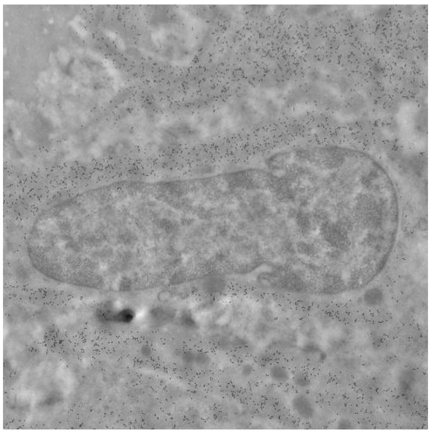

Immunoelectron microscopy analysis of LR white resin-embedded mouse aorta using Actin (GB111364) at dilution of 1: 50.
A goat anti-rabbit antibody preabsorbed with 10nm colloidal gold was used as the secondary antibody, at dilution of 1: 50

Anti -Smad2 Rabbit Polyclonal Antibody

GB11511 100 µL -20℃

Product Information

**Description**	Smad2 rabbit polyclonal
**Protein full name**	Mothers against decapentaplegic homolog 2
**Synonyms**	SMAD2, JV18, V18-1, MADH2, MADR2, hMAD-2, hSMAD family member 2, Mothers against DPP homolog 2, hSMAD2
**Immunogen**	Recombinant protein corresponding to Mouse Smad2
**Isotype**	IgG
**Purity**	Affinity purification
**Predicted MW.**	60KDa
**Observed MW.**	60KDa
**Subcellular location**	Nucleus, Cytoplasm
**Uniprot ID**	Q15796, Q62432, O70436

Applications

applications	species	dilution	Positive tissue
WB	Human, Mouse, Rat	1: 500-1: 1000	heart, liver, brain
IHC	Human, Mouse, Rat	1: 1000	heart, skeletal muscle
IF	Mouse, Rat	1: 500-1: 1000	placenta, skeletal muscle, muscle, inflammatory heart

Background

Receptor-regulated SMAD (R-SMAD) that is an intracellular signal transducer and transcriptional modulator activated by TGF-beta (transforming growth factor) and activin type 1 receptor kinases. Binds the TRE element in the promoter region of many genes that are regulated by TGF-beta and, on formation of the SMAD2/SMAD4 complex, activates transcription. May act as a tumor suppressor in colorectal carcinoma. Positively regulates PDPK1 kinase activity by stimulating its dissociation from the 14-3-3 protein YWHAQ which acts as a negative regulator.

Storage

**Storage**	Store at -20 ℃ for one year. Avoid repeated freeze/thaw cycles.
**Storage Buffer**	PBS with 0.02%sodium azide,100 µg/ml BSA and 50% glycerol.

NOTE: This product is intended for research only.
1.This product is intended for research only.
2.This product is recommended to dilute with the Primary Antibody Dilution Buffer.

Images

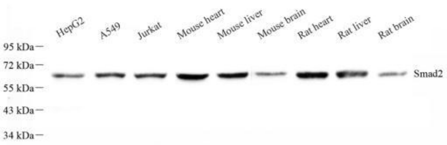

Western blot analysis of Smad2 (GB11511) at dilution of 1:800.

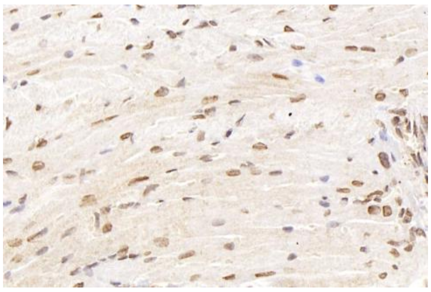

Immunohistochemistry analysis of paraffin-embedded mouse heart using Smad2 (GB11511) at dilution of 1:1000.

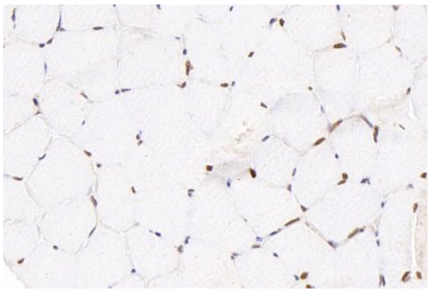

Immunohistochemistry analysis of paraffin-embedded rat skeletal muscle using Smad2 (GB11511) at dilution of 1:1000.

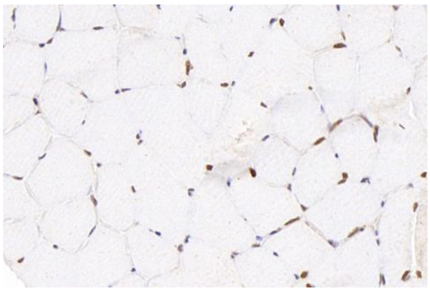

Immunohistochemistry analysis of paraffin-embedded rat skeletal muscle using smad2 (GB11511) at dilution of 1:1000.

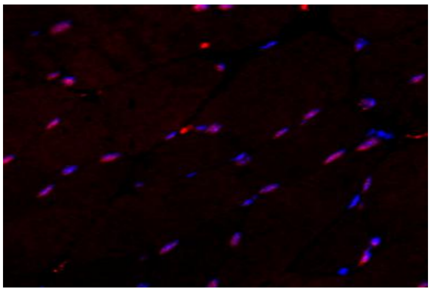

Immunofluorescent analysis of paraformaldehyde-fixed mouse muscle using smad2 (GB11511) at dilution of 1:500.

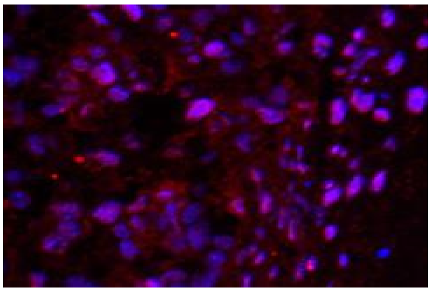

Immunofluorescent analysis of paraformaldehyde-fixed mouse placenta using smad2 (GB11511) at dilution of 1:500.

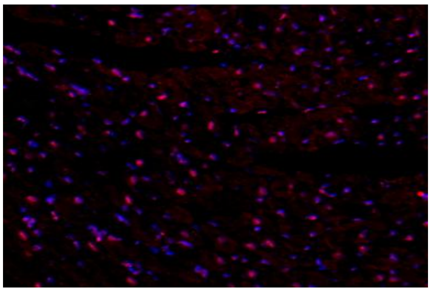

Immunofluorescent analysis of paraformaldehyde-fixed rat inflammatory heart using smad2 (GB11511) at dilution of 1:500.

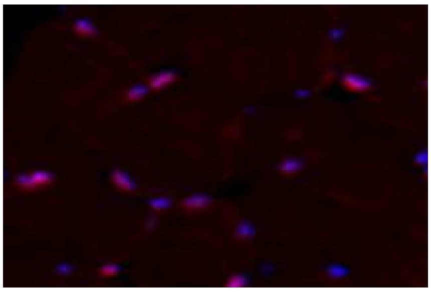

Immunofluorescent analysis of paraformaldehyde-fixed mouse skeletal muscle using smad2 (GB11511) at dilution of 1:1000.

Anti-smad3 Rabbit Polyclonal Antibody

Product Details	
Size	100 µL
Species Reactivity	Human, Mouse, Rat
Host/Isotype	Rabbit / IgG
Class	Polyclonal
Type	Antibody
Conjugate	Unconjugated
Immunogen	Recombinant protein encompassing a sequence within the center region of human SMAD3.
Form	Liquid
Concentration	1 mg/mL
Storage conditions	Store at 4°C short term. For long term storage, store at -20°C, avoiding freeze/thaw cycles.
RRID	AB_2792402

Applications	Tested Dilution	Publications
Western Blot (WB)	1:1,000-1:10,000	-
Immunohistochemistry (Paraffin) (IHC (P))	1:100-1:1,000	-
Immunocytochemistry (ICC/IF)	1:100-1:1,000	-

Product Specific Information

Keep as concentrated solution.

Predicted reactivity: Mouse (100%), Rat (100%), Xenopus laevis (94%), Dog (99%), Pig (100%), Chicken (98%), Rhesus Monkey (99%), Bovine (99%).

Positive Control: HeLa, mouse brain, rat brain, SMAD3-transfected 293T.

Store product as a concentrated solution. Centrifuge briefly prior to opening the vial.

Product Images For SMAD3 Polyclonal Antibody

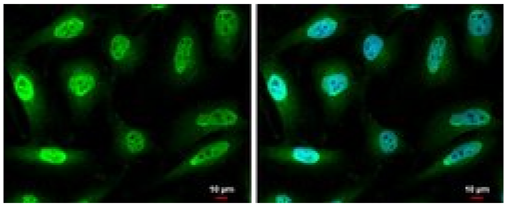

SMAD3 Antibody (PA5-85259) in ICC/IF
Immunocytochemistry analysis of SMAD3 in 4% paraformaldehyde-fixed HeLa cells using SMAD3 polyclonal antibody (Product # PA5-85259) at a dilution of 1:500. Sample was then incubated with Hoechst secondary antibody.

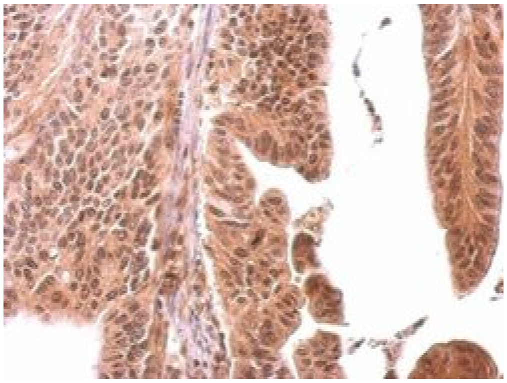

SMAD3 Antibody (PA5-85259) in IHC (P)
Immunohistochemistry analysis of SMAD3 in paraffin-embedded gastric cancer tissue using SMAD3 polyclonal antibody (Product # PA5-85259) at a dilution of 1:500.

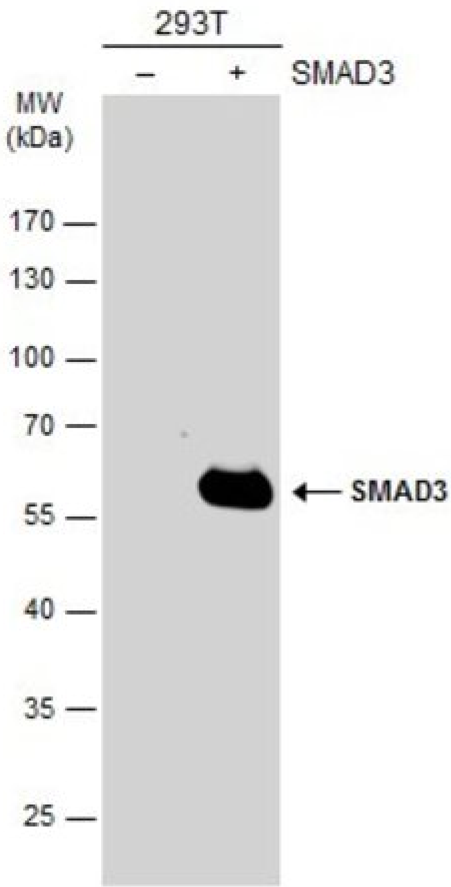

SMAD3 Antibody (PA5-85259) in WB
Western Blot analysis of SMAD3 was performed by separating 30 µg of non-transfected (–) and transfected (+) 293T whole cell extracts by 10% SDS-PAGE. Proteins were transferred to a membrane and probed with a SMAD3 Polyclonal Antibody (Product # PA5-85259) at a dilution of 1:5000. The HRP-conjugated anti-rabbit IgG antibody was used to detect the primary antibody.

For Research Use Only. Not for use in diagnostic procedures. Not for resale without express authorization. Products are warranted to operate or perform substantially in conformance with published Product specifications in effect at the time of sale, as set forth in the Production documentation, specifications and/or accompanying package inserts ("Documentation"). No claim of suitability for use in applications regulated by FDA is made. The warranty provided herein is valid only when used by properly trained individuals. Unless otherwise stated in the Documentation, this warranty is limited to one year from date of shipment when the Product is subjected to normal, proper and intended usage. This warranty does not extend to anyone other than the Buyer. Any model or sample furnished to Buyer is merely illustrative of the general type and quality of goods and does not represent that any Product will conform to such model or sample. NO OTHER WARRANTIES, EXPRESS OR IMPLED, ARE GRANTED INCLUDING WITHOUT LIMITATION, IMPLIED WARRANTIES OF MERCHANTABILITY, FITNESS FOR ANY PARTICULAR PURPOSE, OR NON INFRINGEMENT. BUYER'S EXCLUSIVE REMEDY FOR NON-CONFORMING PRODUCTS DURING THE WARRANTY PERIOD IS LIMITED TO REPAIR, REPLACEMENT OF OR REFUND FOR THE NON-CONFORMING PRODUCT(S) AT SELLER'S SOLE OPTION. THERE IS NO OBLIGATION TO REPAIR, REPLACE OR REFUND FOR PRODUCTS AS THE RESULT OF (I) ACCIDENT, DISASTER OR EVENT OF FORCE MAJEURE, (II) MISUSE, FAULT OR NEGLIGENCE OF OR BY BUYER, (III) USE OF THE PRODUCTS IN A MANNER FOR WHICH THEY WERE NOT DESIGNED, OR (IV) IMPROPER STORAGE AND HANDLING OF THE PRODUCTS. Unless otherwise expressly stated on the Product or in the documentation accompanying the Product, the Product is intended for research only and is not to be used for any other purpose, including without limitation, unauthorized commercial uses, in vitro diagnostic uses, ex vivo or in vivo therapeutic uses, or any type of consumption by or application to human or animals.

Anti-smad4 Rabbit Polyclonal Antibody

Product Details	
Size	100 µL
Species Reactivity	Human, Mouse, Rat
Published Species	Mouse
Host/Isotype	Rabbit / IgG
Class	Polyclonal
Type	Antibody
Conjugate	Unconjugated
Immunogen	Recombinant fragment corresponding to a region within amino acids 322 and 552 of SMAD4 (Uniprot ID#Q13485)
Form	Liquid
Concentration	0.58 mg/mL
Purification	Antigen affinity chromatography
Storage buffer	PBS, pH 7, with 20% glycerol
Contains	0.025% ProClin 300
Storage conditions	Store at 4°C short term. For long term storage, store at -20°C, avoiding freeze/thaw cycles.
RRID	AB_2552158

Applications	Tested Dilution	Publications
Western Blot (WB)	1:500-1:3,000	-
Immunohistochemistry (IHC)	-	1 Publication
Immunohistochemistry (Paraffin) (IHC (P))	1:100-1:1,000	-
Immunocytochemistry (ICC/IF)	1:100-1:1,000	-

Product Specific Information

Recommended positive controls: 293T, A431, HeLa, HepG2, Neuro 2A, C8D30, NIH-3T3, Raw264.7, C2C12, PC-12, Rat2, DDDDK-tagged SMAD4-transfected 293T.

Predicted reactivity: Mouse (100%), Rat (100%), Pig (100%), Sheep (100%), Rhesus Monkey (100%), Bovine (100%).

Store product as a concentrated solution. Centrifuge briefly prior to opening the vial.

Advanced Verification Data

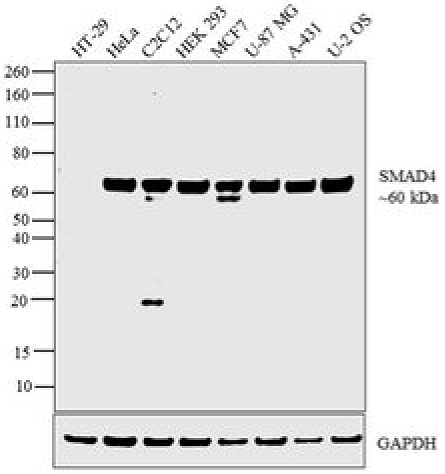

SMAD4 Antibody (PA5-34806)
Antibody specificity was demonstrated by detection of differential basal expression of the target across cell models owing to their inherent genetic constitution. Relative expression of SMAD4 was observed in HT-29 (documented to be a SMAD4 null cell line) when compared to other cell lines tested using product (Product # PA5-34806) in western blot. Relative expression validation info.

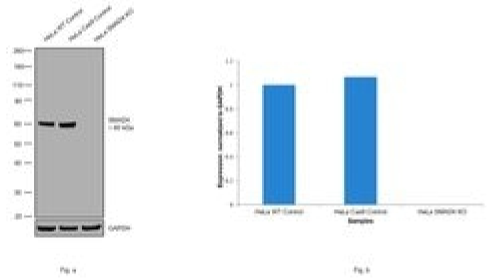

SMAD4 Antibody (PA5-34806)
Antibody specificity was demonstrated by CRISPR-Cas9 mediated knockout of target protein. A loss of signal was observed for target protein in SMAD4 KO cell line compared to control cell line using Anti-SMAD4 Polyclonal Antibody (Product # PA5-34806). Knockout validation info.

1 Reference

Immunohistochemistry (1)

FASEB journal : official publication of the Federation of American Societies for Experimental Biology

Obesity reduces mammary epithelial cell TGF1 activity through macrophage-mediated extracellular matrix remodeling.

"PA5-34806 was used in Immunohistochemistry-immunofluorescence to show TGF1 activity is reduced in the breast epithelial cells in obesity."

Authors: Chamberlin T,Thompson V,Hillers-Ziemer LE,Walton BN,Arendt LM

Species
Mouse

Dilution
1:100

Year
2020

For Research Use Only. Not for use in diagnostic procedures. Not for resale without express authorization. Products are warranted to operate or perform substantially in conformance with published Product specifications in effect at the time of sale, as set forth in the Production documentation, specifications and/or accompanying package inserts ("Documentation"). No claim of suitability for use in applications regulated by FDA is made. The warranty provided herein is valid only when used by properly trained individuals. Unless otherwise stated in the Documentation, this warranty is limited to one year from date of shipment when the Product is subjected to normal, proper and intended usage. This warranty does not extend to anyone other than the Buyer. Any model or sample furnished to Buyer is merely illustrative of the general type and quality of goods and does not represent that any Product will conform to such model or sample. NO OTHER WARRANTIES, EXPRESS OR IMPLED, ARE GRANTED INCLUDING WITHOUT LIMITATION, IMPLIED WARRANTIES OF MERCHANTABILITY, FITNESS FOR ANY PARTICULAR PURPOSE, OR NON INFRINGEMENT. BUYER'S EXCLUSIVE REMEDY FOR NON-CONFORMING PRODUCTS DURING THE WARRANTY PERIOD IS LIMITED TO REPAIR, REPLACEMENT OF OR REFUND FOR THE NON-CONFORMING PRODUCT(S) AT SELLER'S SOLE OPTION. THERE IS NO OBLIGATION TO REPAIR, REPLACE OR REFUND FOR PRODUCTS AS THE RESULT OF (I) ACCIDENT, DISASTER OR EVENT OF FORCE MAJEURE, (II) MISUSE, FAULT OR NEGLIGENCE OF OR BY BUYER, (III) USE OF THE PRODUCTS IN A MANNER FOR WHICH THEY WERE NOT DESIGNED, OR (IV) IMPROPER STORAGE AND HANDLING OF THE PRODUCTS. Unless otherwise expressly stated on the Product or in the documentation accompanying the Product, the Product is intended for research only and is not to be used for any other purpose, including without limitation, unauthorized commercial uses, in vitro diagnostic uses, ex vivo or in vivo therapeutic uses, or any type of consumption by or application to human or animals.

Anti-smad6 Rabbit Polyclonal Antibody

Product Details	
Size	100 µL
Species Reactivity	Human, Mouse
Host/Isotype	Rabbit / IgG
Class	Polyclonal
Type	Antibody
Conjugate	Unconjugated
Immunogen	A synthesized peptide derived from human SMAD6(Accession O43541), corresponding to amino acid residues G363-F389.
Form	Liquid
Concentration	1 mg/mL
Purification	Affinity chromatography
Storage buffer	PBS, pH 7.4, with 50% glycerol, 150mM NaCl
Contains	0.02% sodium azide
Storage conditions	-20°C
RRID	AB_2818334

Applications	Tested Dilution	Publications
Western Blot (WB)	1:500-1:2,000	-
Immunohistochemistry (Paraffin) (IHC (P))	1:50-1:200	-

Product Images For SMAD6 Polyclonal Antibody

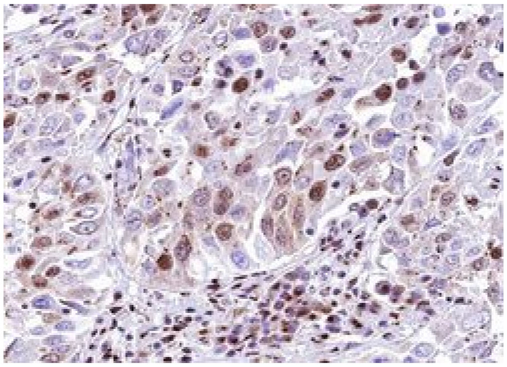

SMAD6 Antibody (PA5-99401) in IHC (P)
Immunohistochemistry analysis of paraffin-embedded SMAD6 in human lung tissue sections. Antigen retrieval was performed using citrate buffer. Samples were blocked with blocking buffer (1.5 hr, 22°C), incubated with SMAD6 polyclonal antibody (Product # PA5-99401) using a dilution of 1:100 (1.5 hr, 22°C), followed by HRP conjugated goat anti-rabbit.

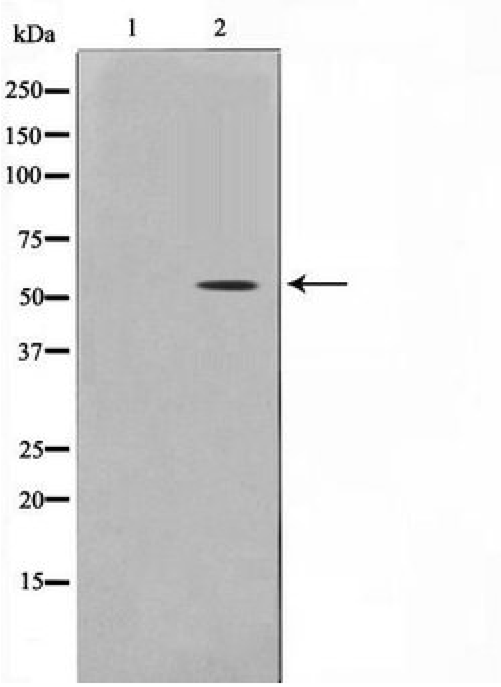

SMAD6 Antibody (PA5-99401) in WB
Western blot analysis of SMAD6 in mouse brain (left lane: treated with the antigen-specific peptide). Samples were incubated with SMAD6 polyclonal antibody (Product # PA5-99401).

For Research Use Only. Not for use in diagnostic procedures. Not for resale without express authorization. Products are warranted to operate or perform substantially in conformance with published Product specifications in effect at the time of sale, as set forth in the Production documentation, specifications and/or accompanying package inserts ("Documentation"). No claim of suitability for use in applications regulated by FDA is made. The warranty provided herein is valid only when used by properly trained individuals. Unless otherwise stated in the Documentation, this warranty is limited to one year from date of shipment when the Product is subjected to normal, proper and intended usage. This warranty does not extend to anyone other than the Buyer. Any model or sample furnished to Buyer is merely illustrative of the general type and quality of goods and does not represent that any Product will conform to such model or sample. NO OTHER WARRANTIES, EXPRESS OR IMPLED, ARE GRANTED INCLUDING WITHOUT LIMITATION, IMPLIED WARRANTIES OF MERCHANTABILITY, FITNESS FOR ANY PARTICULAR PURPOSE, OR NON INFRINGEMENT. BUYER'S EXCLUSIVE REMEDY FOR NON-CONFORMING PRODUCTS DURING THE WARRANTY PERIOD IS LIMITED TO REPAIR, REPLACEMENT OF OR REFUND FOR THE NON-CONFORMING PRODUCT(S) AT SELLER'S SOLE OPTION. THERE IS NO OBLIGATION TO REPAIR, REPLACE OR REFUND FOR PRODUCTS AS THE RESULT OF (I) ACCIDENT, DISASTER OR EVENT OF FORCE MAJEURE, (II) MISUSE, FAULT OR NEGLIGENCE OF OR BY BUYER, (III) USE OF THE PRODUCTS IN A MANNER FOR WHICH THEY WERE NOT DESIGNED, OR (IV) IMPROPER STORAGE AND HANDLING OF THE PRODUCTS. Unless otherwise expressly stated on the Product or in the documentation accompanying the Product, the Product is intended for research only and is not to be used for any other purpose, including without limitation, unauthorized commercial uses, in vitro diagnostic uses, ex vivo or in vivo therapeutic uses, or any type of consumption by or application to human or animals.

Anti-FN Rabbit Polyclonal Antibody

Product Details	
Size	100 µL
Species Reactivity	Human, Mouse, Rat
Published Species	Human
Host/Isotype	Rabbit / IgG
Class	Polyclonal
Type	Antibody
Conjugate	Unconjugated
Immunogen	Recombinant fragment corresponding to a region within amino acids 396 and 689 of Human Fibronectin
Form	Liquid
Concentration	0.14 mg/mL
Purification	Antigen affinity chromatography
Storage buffer	PBS, pH 7, with 20% glycerol, 1% BSA
Contains	0.025% ProClin 300
Storage conditions	Store at 4°C short term. For long term storage, store at -20°C, avoiding freeze/thaw cycles.
RRID	AB_2547054

Applications	Tested Dilution	Publications
Western Blot (WB)	1:500-1:3,000	2 Publications
Immunohistochemistry (IHC)	-	2 Publications
Immunohistochemistry (Paraffin) (IHC (P))	1:100-1:1,000	-
Immunohistochemistry (Frozen) (IHC (F))	Assay-dependent	-
Immunocytochemistry (ICC/IF)	1:100-1:1,000	1 Publication
Flow Cytometry (Flow)	-	1 Publication
Immunoprecipitation (IP)	1:100-1:500	-

Product Specific Information

Recommended positive controls: HepG2, HepG2(10 mM Nicotinamide and 0.4 mM Trichostatin A treatment for 48 hr), HeLa mock and shFN1, mouse plasma.

Predicted reactivity: Mouse (92%), Rat (92%), Chicken (88%), Bovine (92%).

Store product as a concentrated solution. Centrifuge briefly prior to opening the vial.

Advanced Verification Data

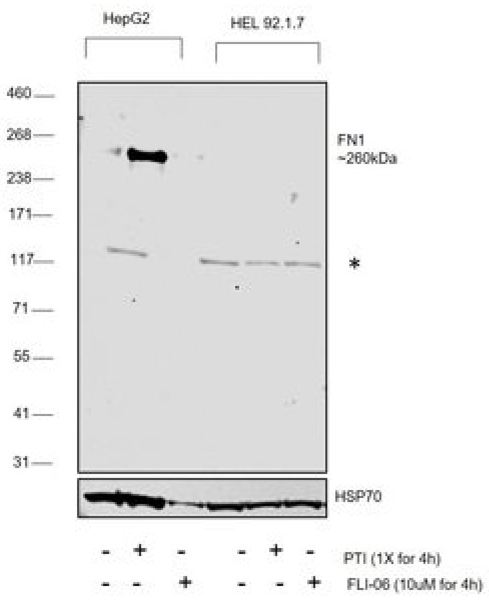

Fibronectin Antibody (PA5-29578)
Altered expression of proteins upon cell treatment demonstrates antibody specificity. Western blot using Fibronectin Polyclonal Antibody (Product # PA5-29578), shows Increased expression of Fibronectin in HepG2 cells upon protein transport inhibitors 1X PTI for 4h and 10uM FLI-06 for 4h versus in HEL 9.2.17 using the same treatments in Western Blot. Another isoform of fibronectin was seen ~120kDa. Cell treatment validation info.

6 References

Western Blot (2)

Scientific reports

Morphological and Molecular Changes in Juvenile Normal Human Fibroblasts Exposed to Simulated Microgravity.

"PA5-29578 was used in Western Blotting to show simulated microgravity causes changes in the cytoskeleton, extracellular matrix, focal adhesion and growth behaviour of normal human dermal fibroblasts and to identify the factors involved in the assembly of the fibroblasts."

Authors: Buken C,Sahana J,Corydon TJ,Melnik D,Bauer J,Wehland M,Krüger M,Balk S,Abuagela N,Infanger M,Grimm D

Species
Human

Dilution
1:1000

Year
2019

Reproductive sciences (Thousand Oaks, Calif.)

1,25 Dihydroxyvitamin D3 Enhances the Antifibroid Effects of Ulipristal Acetate in Human Uterine Fibroids.

"PA5-29578 was used in Western Blotting to suggest that combining vitamin D3 with ulipristal acetate produces additional and orchestrated anti-uterine fibroid effects."

Authors: Ali M,Shahin SM,Sabri NA,Al-Hendy A,Yang Q

Species
Human

Dilution
1:3000

Year
2019

Immunohistochemistry (2)

The Journal of biological chemistry

Diet-induced hepatic steatosis abrogates cell-surface LDLR by inducing *de novo* PCSK9 expression in mice.

"PA5-29578 was used in Immunohistochemistry to examine the effect of hepatic steatosis on LDLR expression and circulating LDL cholesterol levels."

Authors: Lebeau PF,Byun JH,Platko K,MacDonald ME,Poon SV,Faiyaz M,Seidah NG,Austin RC

Species
Human

Dilution
1:200

Year
2019

International wound journal

In vitro assessment of a novel, hypothermically stored amniotic membrane for use in a chronic wound environment.

"Published figure using Fibronectin polyclonal antibody (Product # PA5-29578) in Immunohistochemistry"

Authors: McQuilling JP,Vines JB,Mowry KC

Species
Not Applicable

Dilution
Not Cited

Year
2017

More applications with references on thermofisher.cn

ICC/IF (1) **Flow (1)**

For Research Use Only. Not for use in diagnostic procedures. Not for resale without express authorization. Products are warranted to operate or perform substantially in conformance with published Product specifications in effect at the time of sale, as set forth in the Production documentation, specifications and/or accompanying package inserts ("Documentation"). No claim of suitability for use in applications regulated by FDA is made. The warranty provided herein is valid only when used by properly trained individuals. Unless otherwise stated in the Documentation, this warranty is limited to one year from date of shipment when the Product is subjected to normal, proper and intended usage. This warranty does not extend to anyone other than the Buyer. Any model or sample furnished to Buyer is merely illustrative of the general type and quality of goods and does not represent that any Product will conform to such model or sample. NO OTHER WARRANTIES, EXPRESS OR IMPLED, ARE GRANTED INCLUDING WITHOUT LIMITATION, IMPLIED WARRANTIES OF MERCHANTABILITY, FITNESS FOR ANY PARTICULAR PURPOSE, OR NON INFRINGEMENT. BUYER'S EXCLUSIVE REMEDY FOR NON-CONFORMING PRODUCTS DURING THE WARRANTY PERIOD IS LIMITED TO REPAIR, REPLACEMENT OF OR REFUND FOR THE NON-CONFORMING PRODUCT(S) AT SELLER'S SOLE OPTION. THERE IS NO OBLIGATION TO REPAIR, REPLACE OR REFUND FOR PRODUCTS AS THE RESULT OF (I) ACCIDENT, DISASTER OR EVENT OF FORCE MAJEURE, (II) MISUSE, FAULT OR NEGLIGENCE OF OR BY BUYER, (III) USE OF THE PRODUCTS IN A MANNER FOR WHICH THEY WERE NOT DESIGNED, OR (IV) IMPROPER STORAGE AND HANDLING OF THE PRODUCTS. Unless otherwise expressly stated on the Product or in the documentation accompanying the Product, the Product is intended for research only and is not to be used for any other purpose, including without limitation, unauthorized commercial uses, in vitro diagnostic uses, ex vivo or in vivo therapeutic uses, or any type of consumption by or application to human or animals.
