# Supplementary material for: Activation of the TGF-β1/Smads/α-SMA pathway is related to histological and functional changes in children with neurogenic bladder
Source: Sci Rep. 2022 Jun 3;12:9285. doi: 10.1038/s41598-022-13470-0 (PMC9166803; doi:10.1038/s41598-022-13470-0)
Supplement: Supplementary file 2 — Supplementary Information 2. [file 41598_2022_13470_MOESM2_ESM.pdf]

Activation of the TGF-β1/Smads/α-SMA pathway is related to histological and functional changes in children with neurogenic bladder

Xinghuan Yang[1,2,3], Qingsong Pu[1,2,3], Yibo Wen[1,2,3], Yi Zhao[1,2,3], Junkui Wang[1,2,3], Pengchao Xu[1,2,3], Yuan Ma[1,2,3], Erpeng Liu[1,2,3], Lei Lv[1,2,3] & Jianguo Wen[1,2,3,*]

[1]Pediatric Urodynamic Centre. First Affiliated Hospital of Zhengzhou University, Zhengzhou, China
[2]Henan Joint International Pediatric Urodynamic Laboratory, Zhengzhou, China
[3]Urinary bladder structure and function reconstruction laboratory (Henan Developing and Reform Committee), Zhengzhou, China

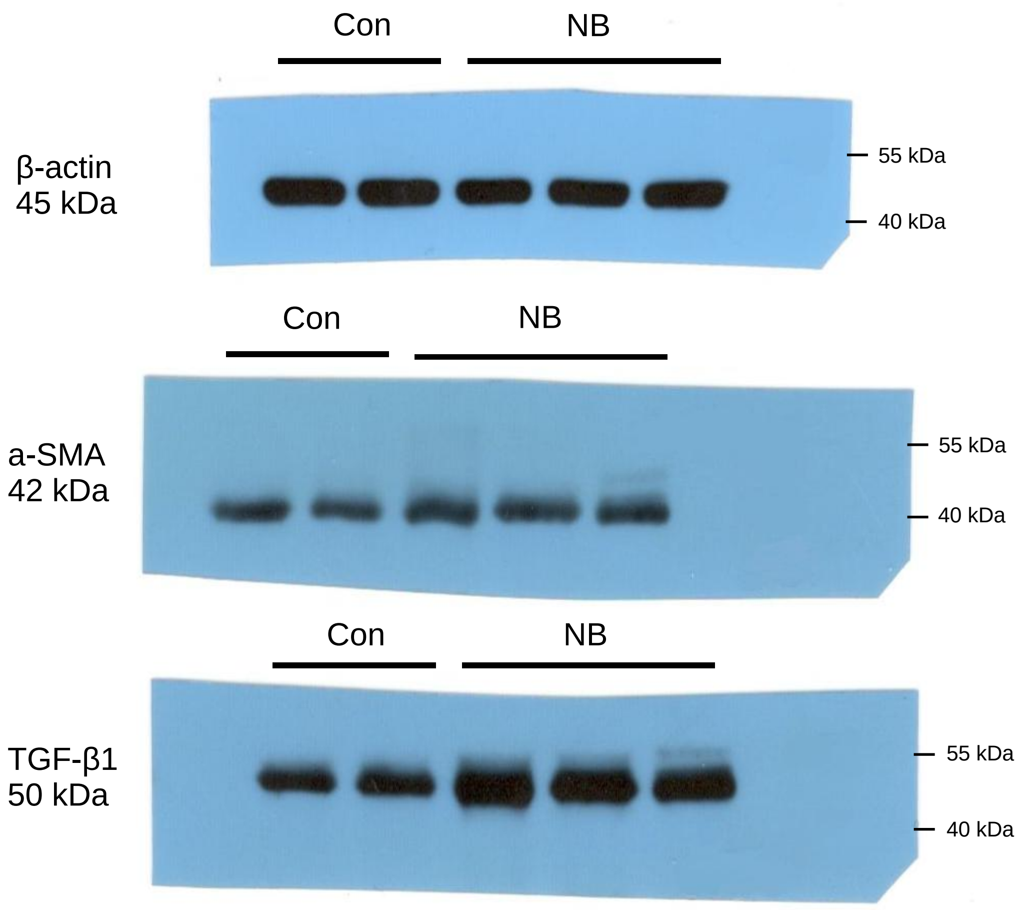

Original gels/blots for Figure. 1a
